# Technical considerations and outcomes in type A aortic dissection with previous cardiac surgery: A narrative review

**DOI:** 10.1016/j.xjon.2026.101841

**Published:** 2026-04-27

**Authors:** Ryaan EL-Andari, John R. Frederick, Sabin J. Bozso

**Affiliations:** a Division of Cardiac Surgery, University of Alberta, Edmonton, Alberta, Canada; b Division of Cardiovascular Surgery, Sanger Heart and Vascular Institute, Charlotte, NC

**Keywords:** aortic dissection, redo sternotomy, reoperation

## Abstract

Previous cardiac surgery increases the technical complexity of ATAAD repair, although it does not result in prohibitive surgical risk for patients presenting with ATAAD. Appropriate patient selection and early establishment of CPB can help to reduce the operative risk associated with ATAAD repair.

**Figure:**
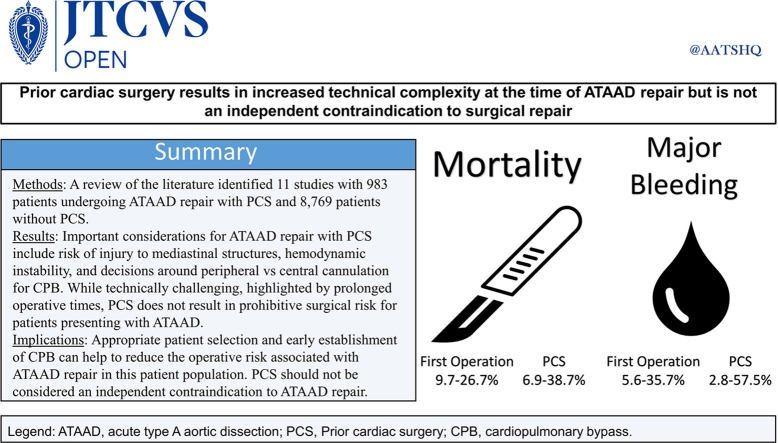

**Figure:**
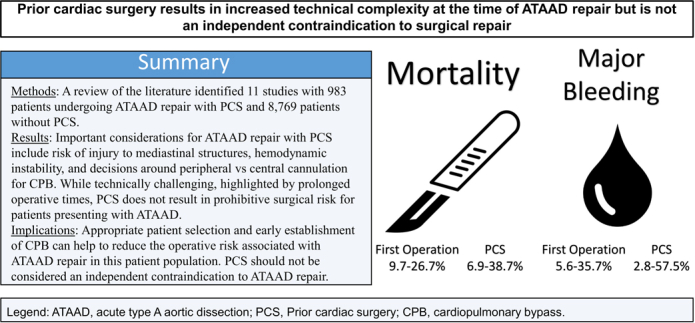
PCS increases the complexity of ATAAD repair but does not result in prohibitive risk.

**Central Message**

PCS should not be considered a contraindication for ATAAD repair in patients with acceptable surgical risk and baseline profile.

**Perspective**

Previous cardiac surgery (PCS) is associated with longer cardiopulmonary bypass time, mixed short-term mortality results, and similar secondary outcomes. Although ATAAD repair in patients with PCS is associated with increased technical complexity, attention to the preoperative CT findings and consideration of early peripheral cannulation for cardiopulmonary bypass can help to reduce operative risk.

Acute type A aortic dissection (ATAAD) is a surgical emergency that requires a technically demanding surgical repair.^1^ In the majority of cases, the challenges associated with surgical repair of ATAAD include hemodynamic instability of the patient, poor tissue quality, planning of cardiopulmonary bypass setup and cerebral protection, and complex aortic arch or root reconstructions.^1^, ^2^, ^3^

In patients who have had previous cardiac surgery (PCS), an additional technical challenge arises in the form of sternal reentry, which is complicated by adhesions, risk of injury to adjacent structures, and increased risk of bleeding, as well as modification of the sequence of the operation requiring consideration of early establishment of cardiopulmonary bypass and dissection of adhesions before aortic repair.^3^, ^4^, ^5^ Challenging sternal reentry, injury to mediastinal structures, and prolonged operative times considerably increase the complexity of ATAAD repair in patients with a previous sternotomy, irrespective of whether the initial cardiac surgery was related to aortic disease or otherwise.^3^^,^^4^^,^^6^^,^^7^ There are limited data available on the technical approaches and outcomes associated with reoperative cardiac surgery for ATAAD. Herein, we review the technical considerations, pitfalls, and outcomes associated with surgical repair of ATAAD in patients with PCS.

## Methods

The PubMed and Embase databases were searched for relevant articles using the following search terms: aortic dissection, type A dissection, reoperation, and reoperative cardiac surgery, individually or in combination. Articles were screened from database inception to the date of the search, July 28, 2025. Articles were included if they reported the outcomes of patients undergoing ATAAD repair comparing patients with and without PCS. The primary search was conducted by one author with a second author approving included studies.

### Pitfalls of Redo Sternotomy in ATAAD Repair

**Figure 1:**
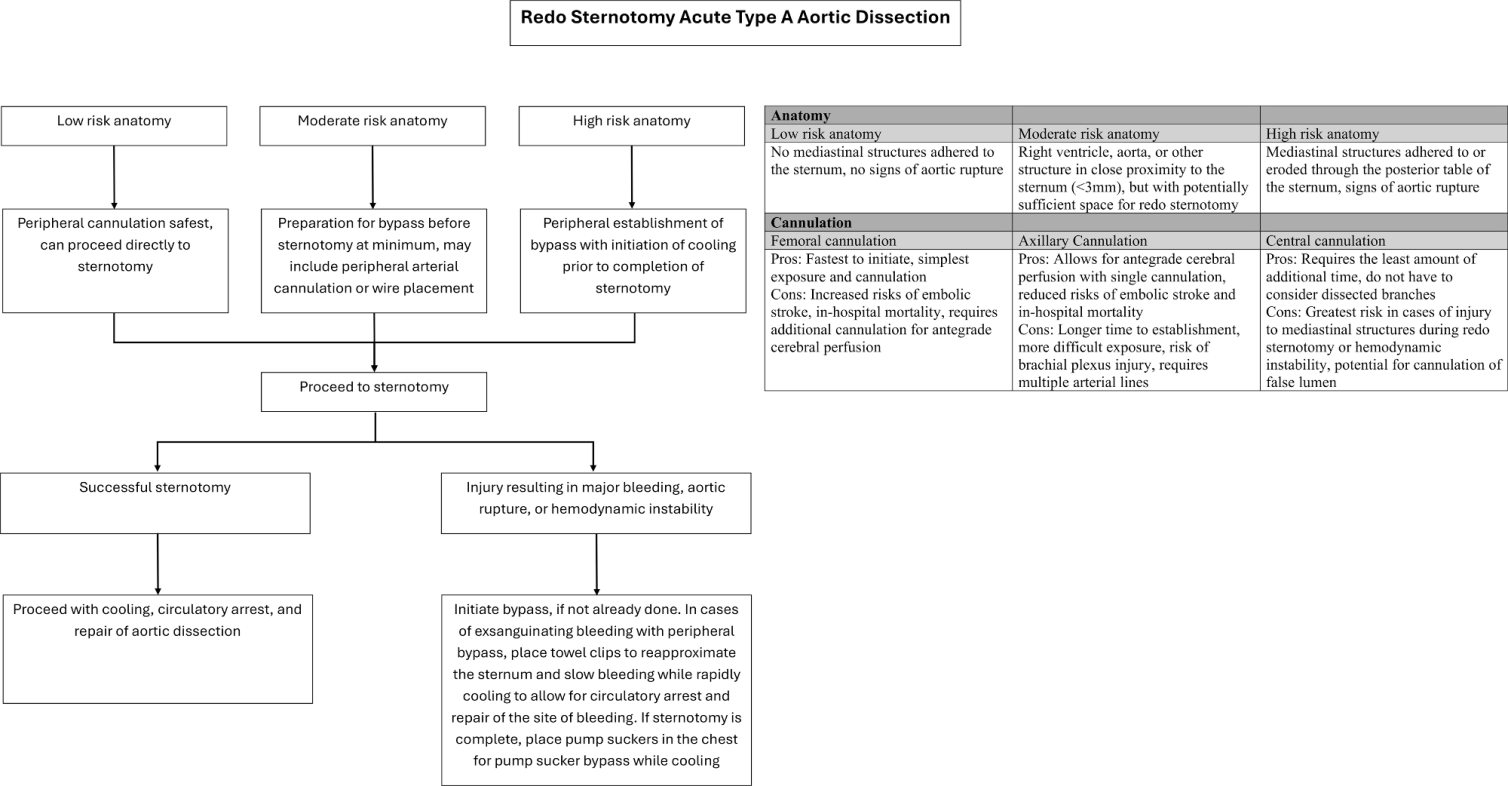
Algorithm for the redo sternotomy in acute type A aortic dissection.

Before reoperation for ATAAD repair in patients with PCS, a computed tomography (CT) scan is necessary to determine structures at risk at the time of redo sternotomy.^1^ Scarring after an initial surgical procedure alters anatomy and may result in closer proximity of important structures to the posterior table of the sternum include the ascending aorta, right ventricle, innominate vein, or any branches originating from the ascending aorta including proximalized head vessels or bypass grafts. Injury to any of the aforementioned structures during sternotomy without established cardiopulmonary bypass may result in life-threatening exsanguination and hemodynamic instability, given the time required to dissect the heart and aorta away from adhesions.^8^^,^^9^ Furthermore, opening of the pericardium in patients with aortic rupture and without peripheral cardiopulmonary bypass may result in life-threatening bleeding. If there is suspicion of aortic rupture, peripheral cardiopulmonary bypass with at least arterial cannulation should be established, such that pump sucker bypass may be initiated in the case of aortic rupture to allow for cooling of the patient and circulatory arrest, however, peripheral cannulation before sternotomy is preferred and safer in ATAAD cases (Figure 1).^1^

### Standard Operative Procedure

Reoperation for patients with PCS may require cannulation for cardiopulmonary bypass before sternotomy. In stable patients with a low risk of injury to mediastinal structures, central cannulation may be performed after completion of redo sternotomy.^3^^,^^10^ In emergent cases with hemodynamic instability or high risk of injury to mediastinal structures attributable to proximity to the sternum, peripheral cannulation is preferred to stabilize the patient and reduce the risk of life-threatening bleeding in the case of vascular or cardiac injury on sternal reentry. Axillary cannulation is commonly used for peripheral cannulation because it provides the ability to provide antegrade cerebral perfusion once the innominate is clamped.^1^^,^^5^^,^^9^^,^^11^ However, axillary cannulation takes time and in patients who are hemodynamically unstable, percutaneous femoral cannulation would provide the fastest option for initiating cardiopulmonary bypass.^11^^,^^12^

### Tips for Safe Sternal Reentry and Surgical Sequence

The first step in ensuring safe sternal reentry is reviewing the preoperative CT scan.^1^ Review of the CT scan should include classification of type and extent of aortic dissection, indications for surgery, as well as at-risk structures during redo sternotomy. In cases in which important structures are close to the posterior table of the sternum, a safe redo sternotomy may still be performed. This can be accomplished by leaving the sternal wires in place to protect mediastinal structures during sternotomy or partially completing the sternotomy with an oscillating saw and dividing the posterior table of the sternum with straight scissors.

Another option for sternal reentry would include partially dividing the sternum and starting at the xiphoid sternum, dissecting mediastinal structures away from the posterior table of the sternum, then dividing that segment of the sternum before repeating this process superiorly until the entire sternum is divided.

In certain cases, such as mediastinal structures directly adhering to or even invading the sternum, it may not be possible to avoid injury to vascular structures.^4^^,^^13^ In these cases, cardiopulmonary bypass is required before sternotomy. Several options exist for peripheral establishment of cardiopulmonary bypass, such as axillary artery cannulation, femoral artery cannulation, and suprasternal innominate artery cannulation, which have all been described and used in various previous publications on reoperative surgery for aortic interventions and other procedures.^1^^,^^3^^,^^8^^,^^9^^,^^11^^,^^12^^,^^14^, ^15^, ^16^ Axillary artery cannulation provides several benefits to femoral artery cannulation, such as antegrade perfusion reducing risk of embolic stroke from retrograde emboli from the aorta, the ability to clamp the innominate artery and continue with antegrade cerebral perfusion, the ability to be recannulated if used in previous procedures, and lower in-hospital mortality and neurologic injury.^1^^,^^8^^,^^12^ An important risk associated with axillary artery cannulation is the brachial plexus, which lies in close proximity to the axillary artery. Care must be taken during dissection and retraction to avoid peripheral nerve injury.^17^

Division of portions of the sternum can be performed during cooling and circulatory arrest should be initiated before completing the division of the sternum, which would result in injury to the underlying vascular structure. With circulatory arrest, there will be no risk of major bleeding; however, there will be a limited amount of time for dissection of adhesions to isolate the innominate artery for antegrade cerebral perfusion. In patients who have been systemically cooled, there is often sufficient time to initiate cerebral perfusion without significant cerebral injury. After the establishment of cardiopulmonary bypass and completion of sternotomy, the heart must be arrested. Clamping the dissected aorta is generally avoided to prevent aortic rupture and embolization of thrombotic material. This limits the feasibility of using antegrade cardioplegia. Placement of a retrograde catheter allows for initial arrest of the heart, after which direct ostial cardioplegia can be delivered once the aorta is opened.^10^ Once these steps are completed, replacement of the aorta may continue. The extent of repair during a reoperation relies on similar principles to a first-time sternotomy aortic dissection repair. In cases of a DeBakey 2 aortic dissection, a standard hemiarch repair is likely all that is required for complete treatment of aortic dissection. In more DeBakey 1 aortic dissections, more extensive repairs such as a total arch with or without frozen elephant trunk may be required in specific cases such as those with malperfusion, branch vessel involvement, aortic arch tears, or aneurysmal aortic arch. However, the surgeon's ability to safely perform an extended arch repair will dictate whether or not this is feasible, with a hemiarch repair being the minimal required intervention if extended arch repairs are not feasible, allowing for an initial life-saving procedure with the opportunity for elective reoperation or endovascular repair if aortic arch repair is required.^9^^,^^16^^,^^18^, ^19^, ^20^

## Results

A literature search was performed for previous publications in which the authors the outcomes of patients undergoing ATAAD repair with or without PCS. Characteristics of the included studies are reported in Table 1,^3^^,^^4^^,^^6^^,^^7^^,^^10^^,^^11^^,^^15^^,^^21^, ^22^, ^23^, ^24^ intraoperative details are reported in Table 2, and postoperative outcomes are reported in Table 3. A total of 11 studies with 8768 patients included 983 patients with PCS undergoing ATAAD repair and 7785 comparison patients without PCS.^3^^,^^4^^,^^6^^,^^7^^,^^10^^,^^11^^,^^15^^,^^21^, ^22^, ^23^, ^24^

**Table 1:** Study characteristics

First author and year	Groups	Years during which data were collected	Patient population	Previous operations (%)
Bjurbom 2023^21^	PCS: 40 No previous surgery: 1119 Total: 1159	2005-2014	Patients from 8 centers in the NORCAAD database with or without previous cardiac surgery undergoing surgical repair for ATAAD. Average age 61.5 y with increased incidence of diabetes, CAD, and greater EuroSCORE in the PCS group.	Valve: 20 (50) (18 aortic valve, 1 mitral valve, 1 aortic + mitral) CABG: 11 (27.5) CABG + valve: 6 (15) (1 aortic, 4 mitral, 1 aortic + mitral) Atrial septal defect closure: 1 (2.5) Ventricular septal defect closure: 1 (2.5) Heart transplantation: 1 (2.5)
Brown 2022^10^	PCS: 72 No previous surgery: 529 Total: 601	2007-2021	Patients with or without previous cardiac surgery undergoing surgical repair for ATAAD at a single center. Average age 61.2-61.4 y with increased incidence of diabetes, hypertension, peripheral vascular disease, atrial fibrillation, and in the PCS group.	CABG: 25 (34.7) CABG + valve: 5 (6.9) Aorta: 13 (18.1) Valve procedure: 12 (16.7) Other: 15 (20.8) (Cox-maze, atrial septal defect repair, pericardiectomy, etc) Unknown: 2 (2.8)
D'Onofrio 2023^3^	PCS: 85 No previous surgery: 1387 Total: 1472	1977-2020	Patients with or without previous cardiac surgery undergoing surgical repair for ATAAD from a multicenter registry including 6 sites. Average age 66 y with increased incidence of atrial fibrillation, CAD, myocardial infarction, stroke, and Marfan syndrome in the PCS group.	AVR: 34 (36.2) CABG: 22 (23.4) Aorta: 21 (22.3) MVR: 7 (7.4) Congenital procedures: 6 (6.4) Other: 4 (4.3)
Estrera 2010^22^	PCS: 49 No previous surgery: 281 Total: 330	1992-2009	Patients with or without previous cardiac surgery undergoing surgical repair for ATAAD at a single center. Average age was 58 in patients without PCS and 63 y in patients with PCS. Patients with and without PCS had comparable baseline comorbidities.	CABG: 30 (61) AVR: 8 (16) CABG + AVR: 5 (10) AVR + mitral valve repair: 1 (2) AVR + tricuspid valve repair: 1 (2) Coarctation repair: 2 (4) Exploration: 1 (2) Congenital procedure: 1 (2)
Klodell 2012^15^	PCS: 31 No previous surgery: 31 Total: 62	1998-2010	Propensity score–matched patients from a single center with or without PCS undergoing surgical repair for ATAAD. Average age was 64.3 y in first cardiac surgery and 66.9 y in PCS with increased rates of current smoking and otherwise comparable baseline demographics after propensity matching. Before propensity matching, patients with PCS had greater rates of COPD, smoking, and atrial fibrillation with increased age.	CABG: 23 (74.2) AVR or root operation: 8 (25.8)
Krebs 2019^23^	PCS: 138 No previous surgery: 1194 Total: 1332	2002-2017	Patients with or without previous cardiac surgery from the STS database undergoing surgical repair for ATAAD. Average age was 58 for no PCS and 63 for PCS. Patients with PCS were more likely to have heart failure, hypertension, peripheral arterial disease, diabetes, and chronic lung disease.	CABG: 56 (46.3) Aortic or mitral valve surgery: 50 (36.2)
Lin 2025^4^	PCS: 74 No previous surgery: 296 Total: 370	2004-2017	1:4 propensity scored matched patients undergoing ATAAD repair with or without PCS from a national database in Taiwan. Average age was 59.2 y in first cardiac surgery and 67.1 y in PCS with comparable baseline demographics following propensity matching. Before propensity matching, patients with PCS were older, had greater rates of diabetes, heart failure, myocardial infarction, stroke, and atrial fibrillation.	Valve: 41 (55.4) CABG: 24 (32.4) Aorta: 21 (28.4) Other including cardiotomy, intracardiac tumor resection, repair of congenital heart defect, repair of ventricular aneurysm, maze procedure and pulmonary embolectomy: 6 (8.1)
Norton 2020^24^	PCS: 50 No previous surgery: 495 Total: 545	1996-2017	Patients with or without previous cardiac surgery undergoing surgical repair for ATAAD at a single center. Average age was 59 for no PCS and 62 for PCS. Patients with PCS were more likely to have coronary artery disease, peripheral arterial disease, smoking history, and connective tissue disorders.	CABG: 15 (30) Aortic valve: 7 (14) Mitral valve: 7 (14) Ascending aortic replacement: 2 (4.0) CABG + AVR: 3 (6.0) CABG + ascending aortoplasty: 1 (2.0) Aortic root replacement + ascending replacement: 8 (16) Multiple valve surgery: 2 (4.0) Multiple valve + ascending aorta: 1 (2.0) Congenital surgery: 2 (4.0) Other: 2 (4.0)
Rylski 2014^7^	PCS: 56 No previous surgery: 573 Total: 629	1993-2013	Patients with or without PCS undergoing surgical repair for ATAAD at a single center. Average age was 60 for no PCS and 70 for PCS. Patients with PCS were more likely to have coronary artery disease.	CABG: 28 (50) AVR: 14 (25) AVR + CABG: 10 (18) MVR + AVR: 1 (2) Congenital: 1 (2) MVR: 1 (2) Heart transplantation: 1 (2)
Teman 2013^6^	PCS: 352 No previous surgery: 1844 Total: 2196	1996-2012	Patients with or without PCS undergoing surgical repair for ATAAD from the IRAD registry including 31 centers from 11 countries. Average age was 61.3 for no PCS and 65.5 for PCS. Patients with PCS were more likely to have hypertension, diabetes, connective tissue disease, and previous aortic dissection.	Aortic surgery (42) Aortic or mitral valve (36) CABG (34) Other (16)
Zhang 2024^11^	PCS: 36 No previous surgery: 36 Total: 72	2015-2023	Propensity score matched patients with or without PCS undergoing surgical repair for ATAAD at a single center. Average age was 56.3 y in first cardiac surgery and 56.9 y in PCS with comparable baseline demographics after propensity matching. Before propensity matching, patients with PCS were older and had greater rates of chronic kidney disease and connective tissue disorders.	Isolated AVR: 16 (43.2) Bentall: 6 (16.2) AVR + MV repair: 3 (8.1) MV replacement/repair: 3 (8.1) AVR + ascending aortoplasty: 2 (5.4) Hemiarch replacement: 2 (5.4) Isolated CABG: 1 (2.7) AVR + CABG: 1 (2.7) David: 1 (2.7) David + HAR: 1 (2.7) Correction of atrial septal defect: 1 (2.7)

*PCS*, Previous cardiac surgery; *NORCAAD*, The Nordic Consortium for Acute Type A Aortic Dissection; *ATAAD*, acute type A aortic dissection; *CAD*, coronary artery disease; *EuroSCORE*, European System for Cardiac Operative Risk Evaluation; *CABG*, coronary artery bypass grafting; *AVR*, aortic valve replacement; *MVR*, mitral valve replacement; *STS*, Society of Thoracic Surgeons; *IRAD*, International Registry of Acute Aortic Dissection; *HAR*, hemiarch repair.

**Table 2:** Intraoperative details for the included studies

First author and year	Groups	Cardiopulmonary bypass time, min, SD/IQR	Crossclamp time, min, SD/IQR	Aortic root procedure, %	Aortic arch procedure, %
Bjurbom 2023^21^	PCS No previous surgery	275 ± 122 205 ± 76, **<.0001**	138 ± 76 105 ± 55, **<.001**	15 (38.5) 285 (25.8), .05	3 (7.7) 63 (5.8)
Brown 2022^10^	PCS No previous surgery	204 ± 84.8 203 ± 72.4, .92		36 (50.0) 195 (36.9), **.032**	11 (15.3) 93 (17.6), .63
D'Onofrio 2023^3^	PCS No previous surgery	266 (192,322) 214 (175,264), **<.001**	136 (89,167) 120 (90,164), .5	18 (21.2) 266 (19.2)	6 (7.1%) 53 (3.8%), .15
Estrera 2010^22^	PCS No previous surgery	226 ± 90.5 166 ± 82.9, **.003**	109 ± 37 99 ± 39, .64		
Klodell 2012^15^	PCS No previous surgery	276.7 (97.4) 204.6 (54.4), **.001**	159.2 (60.1) 129.2 (57.3), .055		
Krebs 2019^23^	PCS No previous surgery	217.5 (150-293) 177 (140-222), **<.01**	110 (83.5-150.5) 104 (77-145), .59	42 (30.4) 441 (36.9), .13	33 (23.9) 269 (30.9), .09
Lin 2025^4^	PCS No previous surgery			10 (13.5) 35 (11.8), .66	13 (17.6) 61 (20.6), .49
Norton 2020^24^	PCS No previous surgery	269.5 (216-326) 219 (177-267), **<.001**	187.5 (137-239) 154 (115-198), **.002**	36 (72) 355 (92), **<.001**	16 (32) 175 (35.8), .26
Rylski 2014^7^	PCS No previous surgery	258 (232-281) 209 (183-247), **<.001**	179 (150-211) 140 (117-168), **<.001**	50 (89) 565 (98.6)	3 (5) 24 (4), .947
Teman 2013^6^	PCS No previous surgery	235.5 (205.3-295.5) 191.0 (150.0-235.0), **<.001**	165.5 (65-182.8) 118.0 (72.0-162.0), .11	88 (39) 503 (35), .23	
Zhang 2024^11^	PCS No previous surgery	210 (188-239) 188 (160-209), **.018**	103 (91-130) 115 (82-131), .724	8 (22.2) 10 (27.7)	36 (100) 36 (100)

Bolded values indicate statistical significance. *SD*, Standard deviation; *IQR*, interquartile range; *PCS*, previous cardiac surgery.

**Table 3:** Outcomes of included studies

First author and year	Groups	In-hospital/30-day mortality, n (%)	Mortality at longest follow-up	ICU length of stay, d	Hospital length of stay, d	Stroke, n (%)	Major bleeding, n (%)
Bjurbom 2023^21^	PCS No previous surgery	12 (30) 199 (17.8) **.049**		7 (3-15) 4 (2-7), **.018**		9 (23.1) 168 (15.4), .19	23 (57.5) 399 (35.7), **.005**
Brown 2022^10^	PCS No previous surgery	5 (6.9) 62 (11.7), .23	28.4% 25.5%		11.4 ± 7.82 12.4 ± 13.4, .55	1 (1.4) 25 (4.7), .35	4 (5.6) 50 (9.5), .38
D'Onofrio 2023^3^	PCS No previous surgery	20 (24) 249 (18), .8	20 y 68% 69.8%, .37	4 (2, 7) 4 (2, 8), >.9	17 (11, 23) 14 (9, 22), .8	8 (11) 183 (14), >.9	33% 16%, **.004**
Estrera 2010^22^	PCS No previous surgery	15 (31) 39 (13.8), **.007**	15 y 64% 47%, .07			5 (10) 7 (2.5), **.03**	2 (4) 24 (8.5), .39
Klodell 2012^15^	PCS No previous surgery	12 (38.7) 3 (9.7), **.008**		19.4 (34.9) 14.8 (13.7), .61	23.2 (36.6) 20.1 (15.9), .35	2 (7.1) 7 (22.6), .15	6 (21.4) 3 (9.7), .29
Krebs 2019^23^	PCS No previous surgery	39 (28.3) 183 (15.3), **<.01**		5.7 (2.8-10.7) 4.8 (2.6-9.9), .37	13 (8-20) 10 (6-17), **.02**	9 (6.6) 113 (9.5), .26	13 (9.4) 81 (6.8), .25
Lin 2025^4^	PCS No previous surgery	20 (27.0) 76 (26.7), .95	5 y 29.6% 23.1%, .31		29.2 ± 27.9 22.2 ± 37.6, .19	3 (4.1) 13 (4.4), .83	5 (6.8) 22 (7.4), .95
Norton 2020^24^	PCS No previous surgery	6 (12) 38 (7.7), .28	10 y 34% 20%, **.02**		14.5 (9-28) 11 (7-18), **.02**	1 (2.0) 37 (7.5), .24	7 (14) 40 (8.1), .18
Rylski 2014^7^	PCS No previous surgery	14 (25) 69 (12), **.011**	10 y 62% 50%, **.003**			2 (4) 30 (5), .82	
Teman 2013^6^	PCS No previous surgery	118 (34) 432 (23), **<.001**	2 y 45 15%, **<.001**			18 (6) 159 (10), **.046**	
Zhang 2024^11^	PCS No previous surgery	5 (13.9) 4 (11.1), >.99		6 (2-10) 5 (3-7), .29	14 (9-18) 12 (8-14), .062		1 (2.8) 2 (5.6), >.99

Bolded values indicate statistical significance. *PCS*, Previous cardiac surgery.

### Intraoperative Details

Cardiopulmonary times tended to be longer for patients with PCS with all studies except one (Table 2).^3^^,^^6^^,^^7^^,^^11^^,^^15^^,^^21^, ^22^, ^23^, ^24^ In contrast, crossclamp time was comparable between patients with and without PCS in 8 of 11 studies, suggesting that the extra operative time was likely required for presternotomy cannulation and dissection of adhesions, rather than the aortic replacement itself.^3^^,^^6^^,^^7^^,^^10^^,^^11^^,^^15^^,^^21^, ^22^, ^23^, ^24^ Similar rates of aortic root and aortic arch procedures were reported in most studies (Table 2).^3^^,^^4^^,^^6^^,^^7^^,^^11^^,^^15^^,^^21^, ^22^, ^23^

### Mortality

In-hospital or 30-day mortality was reported in all studies. Six studies reported significantly greater rates of mortality for patients with PCS (28.3-38.7%) compared with primary sternotomy (9.7-23%, differences ranging from 11.0% to 19.0%, *P* = .049-<.001) (Table 3).^6^^,^^7^^,^^15^^,^^21^, ^22^, ^23^ In contrast, the remaining 5 studies did not find significant differences in in-hospital mortality between patients with PCS (6.9-27.0) and patients without PCS (7.7-26.7, differences 0.3-6.0%, *P* = .23-1.0) (Table 3).^3^^,^^4^^,^^10^^,^^11^^,^^24^

Five-year mortality was reported in the study by Lin and colleagues,^4^ who found rates of mortality to be 29.6% for patients with PCS and 23.1% for patients without (*P* = .31) (Table 3). Similarly, Brown and colleagues^10^ did not find significant differences in mortality at a median follow-up time of 4.6 years (28.4% vs 25.5%). Ten-year mortality was greater in the PCS group in the studies by Norton and colleagues^24^ (34.0% vs 20.0%, *P* = .02) and Rylski and colleagues^7^ (62.0% vs 50.0%, *P* = .003), whereas longer-term follow-up of 15 and 20 years in the studies by Estrera and colleagues^22^ (64.0% vs 47.0%, *P* = .07) and D'Onofrio and colleagues^3^ (68.0% vs 69.8%, *P* = .37), respectively, did not have significantly different survival (Table 3).

### Stroke

Postoperative strokes were comparable between patients with and without PCS in all studies but two. Rates of stroke in the included studies for PCS versus non-PCS were 23.1% versus 15.4% (*P* = .19), 1.4% versus 4.7% (*P* = .35), 11.0% versus 14.0% (*P* > .9), 7.1% versus 22.6% (*P* = .15), 6.6% versus 9.5% (*P* = .26), 4.1% versus 4.4% (*P* = .83), 2.0% versus 7.5% (*P* = .24), and 4.0% versus 5.0% (*P* = .82) (Table 3).^3^^,^^4^^,^^7^^,^^10^^,^^11^^,^^15^^,^^21^^,^^23^^,^^24^ The 2 studies in which authors found a significant difference in rates of stroke were Teman and colleagues,^6^ who found fewer strokes in patients with PCS (6%) compared with patients without PCS (10%) (*P* = .046), and Estrera and colleagues,^22^ who found stroke rates of 10% for the PCS group and 2.5% for the no previous surgery group (*P* = .03) (Table 3).

### Major Bleeding

In 9 of 11 studies comparing patients with PCS and those without, there was no significant difference in major bleeding between the groups (*P* = .18-.99) (Table 3).^4^^,^^9^, ^10^, ^11^^,^^22^, ^23^, ^24^ Rates of major bleeding in the included studies for PCS versus non-PCS were 5.6% versus 9.5% (*P* = .38), 4.0% versus 8.5% (*P* = .39), 21.4% versus 9.7% (*P* = .29), 9.4% versus 6.8% (*P* = .25), 6.8% versus 7.4% (*P* = .95), 14.0% versus 8.1% (*P* = .18), and 2.8% versus 5.6% (*P* > .99). In 2 studies, the authors found greater rates of major bleeding in patients with PCS (33.0% vs 16.0%, *P* = .004 and 35.7% vs 57.5%, *P* = .005) (Table 3).^3^^,^^21^

### Hospital Length of Stay (LOS)

Intensive care unit (ICU) and hospital LOS were reported in 9 studies and varied considerably in LOS between studies. LOS was comparable between groups in most studies (Table 3). Bjurbom and colleagues^21^ reported a longer average ICU LOS in patients with PCS at 7 days compared with 4 days for patients without PCS. Overall hospital LOS was reported to be longer for patients with PCS in the studies by Lin and colleagues,^4^ 13 days for PCS versus 10 days for no PCS (*P* = .02), and Norton and colleagues,^24^ 14.5 days for PCS versus 11 days for no PCS (*P* = .02). In the remaining 6 studies, authors found no significant differences in either ICU or hospital LOS (ICU PCS vs non-PCS 4 days vs 4 days, 19.4 days vs 14.8 days [*P* = .61], 5.7 days versus 4.8 days [*P* = .37], 6 days versus 5 days [*P* = .29]) (hospital PCS vs non-PCS 11.4 days vs 12.4 days [*P* = .55], 17.0 days versus 14.0 days [*P* = .80], 23.2 days versus 20.1 days [*P* = .35], 29.2 days versus 22.2 days [*P* = .19], and 14.0 days versus 12.0 days [*P* = .06]) (Table 3).

## Discussion

PCS is an important consideration for patients presenting with ATAAD. A previous sternotomy increases the difficulty of the operation, increases the time taken to establish central cardiopulmonary bypass in unstable patients, and increases surgical risk. Thus, PCS has previously been considered a relative contraindication or risk factor for ATAAD repair, especially in patients who are not optimal surgical candidates, because they often present with elevated baseline surgical risk, advanced age, or significant comorbidities that increase surgical risk.^4^^,^^6^^,^^11^^,^^15^^,^^21^^,^^23^, ^24^, ^25^^,^^E1^ However, with increased experience at high-volume centers, there is evidence to suggest that ATAAD repair in patients with PCS is not only feasible but carries acceptable risk in the context of a complex ATAAD repair.^5^^,^^10^^,^^18^ Most studies found no differences in stroke with 2 studies, with the authors of 1 large multicenter registry finding lower rates of stroke with PCS and the authors of a smaller single-center study finding greater rates of stroke with PCS.^6^^,^^22^ Mortality was greater in a subset of studies that tended to include smaller sample sizes and higher rates of comorbidities for the PCS group.^6^^,^^15^^,^^21^, ^22^, ^23^ Studies with larger cohorts of patients with PCS, comparable baseline comorbidities, or propensity score matching tended to report similar rates of short-term mortality.^3^^,^^4^^,^^10^^,^^11^

Despite elevated rates of mortality in a subset of studies, the overall risk to patients is not prohibitive. This is likely attributable to multiple factors, including the presence of adhesions protecting patients with aortic rupture from severe hemorrhage, a selection bias in which patients presenting with ATAAD and a PCS may need to have a greater likelihood of survival before proceeding with surgical repair compared with patients presenting for primary sternotomy, and heterogeneity between studies in terms of patient inclusion and surgical procedures. Although redo sternotomy in the setting of ATAAD may be challenging, several key steps are imperative for improving the safety of the operation.

Patient selection is central to the success of a prolonged reoperation. Patients with extensive comorbidities, especially baseline frailty, carry a greater risk of morbidity and mortality, and they may require a longer recovery period after a challenging operation, such as a redo sternotomy with extensive aortic repair.^4^^,^^10^^,^^11^^,^^15^^,^^23^^,^^25^^,^^E1^ In patients with high surgical risk, endovascular treatment of ATAAD has been performed with ongoing trials such as the ARISE trials.E2, E3 These cases are reserved for high-risk surgical patients with appropriate anatomy. Required anatomy to facilitate endovascular treatment of ATAAD includes adequate proximal and distal landing zones, head vessels that are amenable to endovascular treatment (appropriate size and length), and the absence of aortic root involvement requiring intervention, however, endo-Bentall procedures have been reported in select case reports.E2, E3, E4

A detailed review of the preoperative CT scan will allow the surgeon to determine the risk associated with redo sternotomy and the structures at risk during sternal reentry. This will allow the surgeon to avoid mediastinal structures that are near the posterior table of the sternum or to modify the surgical plan with peripheral cannulation for cardiopulmonary bypass allowing for cooling before sternotomy, rapid initiation of bypass should a vascular structure be injured, or stabilization of an unstable patient. The type of previous surgery has considerable implications for the reoperative risk, with coronary artery bypass grafting being the most common PCS and patent coronary bypass grafts posing a considerable risk if they lie close to the posterior table of the sternum.^23^ Notably, in the majority of studies, cardiopulmonary bypass time was significantly longer with PCS whereas crossclamp time was not. This likely reflects the need for peripheral cannulation and time for sternal reentry, with similarity in the aortic portion of the operation once sternotomy and dissection of adhesions is complete.

On the basis of available evidence, ATAAD repair in patients with PCS can be performed with an acceptable risk in well-selected patients. PCS should not be considered an independent contraindication for ATAAD repair but certainly requires consideration in the context of the patient presentation and overall comorbidities.

### Limitations

There are several limitations to this review. Although a significant number of studies were identified, the majority reported retrospective experiences, small samples sizes, and infrequent risk adjustment. Furthermore, the search conducted in this study was meant to be identify representative studies rather than being systematic, resulting in some potentially missed studies in the literature. Finally, heterogeneity between the studies in terms of era surgery was performed in, previous operations, and patient selection impacts the ability to directly compare results between studies.

## Conclusions

ATAAD repair in patients with PCS is associated with increased technical complexity, given the challenging sternal reentry in an already-complex case, oftentimes complicated by hemodynamic instability. Attention to the preoperative CT scan findings and consideration of early peripheral cannulation for cardiopulmonary bypass can help to reduce operative risk to patients, allowing for mitigation of risk during sternal reentry and a relatively safe reoperation.

## Conflict of Interest Statement

The authors reported no conflicts of interest.

The *Journal* policy requires editors and reviewers to disclose conflicts of interest and to decline handling or reviewing manuscripts for which they may have a conflict of interest. The editors and reviewers of this article have no conflicts of interest.
